# HER2 Interactome Profiling Reveals MARCKS as a Candidate Marker Associated with Aggressive Breast Cancer

**DOI:** 10.3390/cancers17172882

**Published:** 2025-09-02

**Authors:** Atsushi Yokoyama, Shun Sawatsubashi, Akiko Ebata, Yasuhiro Miki, Yuri Otsubo, Takashi Suzuki

**Affiliations:** 1 Department of Molecular Endocrinology, Tohoku University Graduate School of Medicine, 2-1 Seiryo-machi, Aoba-ku, Sendai 980-8575, Japan; yuri.otsubo.q2@dc.tohoku.ac.jp; 2 Research and Innovation Liaison Office, Institute of Advanced Medical Sciences, Tokushima University, 3-18-15 Kuramoto-cho, Tokushima 770-8503, Japan; 3 Department of Breast and Endocrine Surgical Oncology Graduate School of Medicine Tohoku University, 1-1 Seiryo-machi, Aoba-ku, Sendai 980-8574, Japan; akiko.ebata.d2@tohoku.ac.jp; 4 Department of Anatomic Pathology, Tohoku University Graduate School of Medicine, 2-1 Seiryo-machi, Aoba-ku, Sendai 980-8575, Japantakashi.suzuki.c1@tohoku.ac.jp (T.S.)

**Keywords:** HER2, RIME, breast cancer, MARCKS, interactome, proteomics, Mass Spectrometry, TCGA, biomarker

## Abstract

**Background/Objectives:** HER2, a critical diagnostic marker and therapeutic target in breast cancer, is a membrane receptor that forms diverse signaling complexes, the constituents of which have not been fully characterized in actual breast cancer tissues. **Methods:** In this study, we applied the Rapid Immunoprecipitation Mass Spectrometry of Endogenous Proteins (RIME) method, originally developed to explore transcription factor complexes, to identify the complexes formed by HER2 in HER2-positive breast cancer specimens. **Results:** Through our approach, we successfully identified multiple complex components, including MARCKS, a novel HER2-interacting partner, which we verified using both proximal ligation assay in cultured cells and immunohistochemistry in tissue sections. TCGA analysis further revealed that high MARCKS expression significantly correlates with ER negativity, as confirmed by multivariate analysis, suggesting its potential role as a prognostic indicator in aggressive breast cancer subtypes. **Conclusions:** These results demonstrate the capability of RIME to elucidate interactomes of membrane proteins such as HER2 in clinical tissue specimens. Furthermore, this study highlights its broader applicability beyond nuclear proteins, underscoring its potential for discovering novel prognostic and diagnostic clinical markers in diverse cancer types.

## Simple Summary

HER2 is a protein found on the surface of breast cancer cells and is important for determining treatment strategies. Drugs that target HER2, such as trastuzumab, have greatly improved the outcomes of patients with HER2-positive breast cancer. However, these treatments do not work for all patients, and we still do not fully understand how HER2 functions in real human tumors. In this study, we used a method called RIME (Rapid Immunoprecipitation Mass Spectrometry of Endogenous Proteins) to analyze HER2-related proteins directly from breast cancer tissue samples. We identified many proteins that interact with HER2, including one called MARCKS, which is known to be involved in aggressive types of cancer. MARCKS was more commonly found in breast cancers that do not respond to hormone therapy, suggesting its potential as a prognostic marker for aggressive tumors. Our study shows that RIME can be used to study HER2-related proteins in clinical samples, and it may help identify a novel prognostic indicator for breast cancer patients.

## 1. Introduction

Breast cancer classification and treatment strategies heavily rely on the expression status of estrogen receptor (ER), progesterone receptor (PR), and human epidermal growth factor receptor 2 (HER2) [1,2,3]. ER and PR, as key nuclear hormone receptors, are essential for forecasting the effectiveness of endocrine therapies [4]. ER-positive breast cancers, which constitute the majority of hormone receptor-positive cases, are typically treated with selective estrogen receptor modulators (e.g., tamoxifen) or aromatase inhibitors to inhibit estrogen-driven tumor growth. PR, as a downstream target of ER signaling, serves as an additional prognostic marker, refining predictions of treatment response [5].

HER2, a transmembrane receptor tyrosine kinase that belongs to the EGFR family, plays a pivotal role in oncogenic signaling. This receptor family, which consists of EGFR, HER2/ERBB2, ERBB3, and ERBB4, mediates cell proliferation and survival through key pathways such as PI3K/AKT and MAPK [6,7]. HER2 is unique among these receptors in that it lacks a known ligand and is constitutively active upon dimerization with other EGFR family members [8,9,10]. Overexpression or amplification of HER2 occurs in approximately 20–25% of breast cancers and is associated with aggressive tumor behavior and poor prognosis [11,12,13]. This overexpression leads to uncontrolled cell growth and division, necessitating the development of HER2-targeted therapies, which have dramatically transformed the treatment landscape. The introduction of HER2-targeted therapies, including monoclonal antibodies such as trastuzumab and pertuzumab, has significantly improved clinical outcomes for HER2-positive breast cancer patients by directly inhibiting HER2-mediated signaling [14,15].

The complexity of HER2 protein interactants within various subtypes of breast cancer significantly influences tumor behavior, treatment responses, and prognostic outcomes [16,17,18]. The variability in the HER2 complex composition, including its co-receptors and associated signaling proteins, modulates tumor aggressiveness and the efficacy of targeted therapies [19,20,21]. This underscores the critical need for a deeper understanding of the HER2 interactome in each subtype of breast cancer, which could reveal novel prognostic markers for precision medicine and inform strategies for assessing disease progression. However, comprehensive characterization of the HER2 interactome in human breast cancer tissues remains challenging, underscoring the need for innovative approaches to elucidate its functional landscape.

Although several studies have investigated the HER2 interactome, all have relied on cultured cells, and none have explored the native interactome of HER2 within breast cancer specimens. The RIME (rapid immunoprecipitation mass spectrometry of endogenous proteins) method, originally developed to analyze the interactome of endogenous transcription factors, employs formalin fixation to stabilize protein interactions, followed by immunoprecipitation with antibodies against endogenous proteins and identification via mass spectrometry [22,23]. This technique not only identifies stable interaction partners but also allows for the detection of weaker or transiently binding factors [24,25,26]. Given the broad applicability of the RIME method, recent studies have also reported its use in analyzing the estrogen receptor interactome from pathological samples [27]. Considering its potential beyond nuclear proteins, we investigated the feasibility of using the RIME method to analyze the HER2 interactome directly from breast cancer pathological specimens.

Therefore, in the present research, we aimed to establish a method for the direct identification of the HER2 interactome from HER2-positive breast cancer specimens using the RIME technique and to assess its potential for identifying novel interactants that could serve as diagnostic and prognostic markers.

## 2. Materials and Methods

### 2.1. Cell Culture

SK-BR-3 cells and MCF-7 cells were obtained from obtained from the American Type Culture Collection and maintained in RPMI 1640 medium (Fujifilm Wako Pure Chemical, Osaka, Japan) supplemented with 10% fetal bovine serum (Nichirei Biosciences, Tokyo, Japan), 100 U/mL penicillin G and 100 μg/mL streptomycin (Fujifilm Wako Pure Chemical) at 37 °C in 5% CO_2_.

### 2.2. Clinical Specimens

Two cases of invasive ductal carcinoma specimens were obtained from female patients who underwent surgical treatment at Tohoku University Hospital (Sendai, Japan) in 2011. Case 1 (TH-BC86) was a 42-year-old patient with pT2, pN1, cM0, Stage IIB, ER positive (TS3), and HER2 score 3. Case 2 (TH-BC83) was a 55-year-old patient with pT1c, pN0, cM0, Stage IA, ER positive (TS3), and HER2 score 3. Case 3 (TH-BC62) was 74-year patient with pT1c, pN0(sn), cM0, Stage I, ER negative. The study was reviewed and approved by the Ethical Committee of Tohoku University School of Medicine (2019-1-311).

### 2.3. Tissue Lysate Preparation from Clinical Breast Cancer Material

Clinical specimens were cryosectioned in 30 µm slices using the CM 3050 S cryostat (Leica, Wetzlar, Germany). In total, 15 slices of tissue sections were fixed in 1% FA for 20 min. Crosslinking was quenched by the addition of glycine to a final concentration of 250 mM. Samples were centrifuged for 3 min at 2500× *g,* and the supernatants were discarded. Tissue pellets were washed twice with cold PBS and resuspended in 6 mL LB3 buffer (10 mM Tris-HCl (pH 8), 100 mM NaCl, 1 mM EDTA, 0.5 mM EGTA, 0.1% Na-deoxycholate, and 0.5% N-lauroylsarcosine), followed by sonication using ultrasonic disrupter (UR-21P, TOMY, Tokyo, Japan) for 8 cycles (level 8, 20 s). After adding 600 μL of 10% Triton X-100, samples were centrifuged for 10 min at 22,000× *g,* and supernatants were used for further analysis.

### 2.4. Rapid Immunoprecipitation Mass Spectrometry of Endogenous Proteins (RIME)

For RIME experiments, 20 μL of Dynabeads Protein G (Thermo Fisher Scientific, Norristown, PA, USA) and 5 μg of specific antibody were used for each sample. The antibodies used were as follows: Anti-ErbB2/HER2 antibody [3B5] (16901, Abcam, Cambridge, UK), Anti-Human c-erbB-2 Oncoprotein (A0485, DAKO, Glostrup, Denmark), Trastuzmab (kindly provided by Setsurotech, Tokushima, Japan), and mouse IgG antibody (sc-2025, Santa Cruz, CA, USA). The samples resuspended in LB3 buffer were incubated with the bead-bound antibody overnight at 4 °C. The next day, the beads were washed 10 times with 1 mL ice-cold RIPA buffer and twice with 500 μL 100 mM AMBIC (ammonium bicarbonate). Washed beads were directly digested with 10 μL of 10 ng/μL Trypsin Gold (Promega, Madison, WI, USA) and incubated at 37 °C overnight. The next day, 10 μL of Trypsin solution was added and incubated 4 h at 37 °C. Digested peptides were cleaned up using the Pierce Detergent Removal Spin Column (Thermo Fisher Scientific, Pittsburgh, PA, USA) and GL-Tip SDB (GL Sciences, Tokyo, Japan), followed by LC-MS/MS analysis using the Orbitrap Fusion (Thermo Fisher Scientific). For protein identification and label-free quantification, spectra were processed using Proteome Discoverer, version 2.5 (Thermo Fisher Scientific) with the Mascot algorithm against the human protein database from SwissProt. Raw data were deposited in the jPOST Repository (JPST003490, JPST003671, and JPST003937).

### 2.5. LC-MS/MS Analysis

Acquired raw data were analysed against the Swiss Prot database restricted to Homo sapiens using Proteome Discoverer version 2.5 (Thermo Fisher Scientific) with the Mascot algorithm. Peptides were filtered at a false discovery rate (FDR) of 1% using the Percolator node. Label-free quantification was performed based on the intensities of the precursor ions using a precursor ion quantifier node. Proteins with statistically significant modification levels were identified using the ANOVA (*p*  <  0.05) between HER2 triplicates and IgG negative control triplicates.

### 2.6. Proximity Ligation Assay (PLA)

SK-BR-3 cells were fixed in 10% formalin and permeabilized with 0.1% Triton X-100. Then, PLA was performed using Duolink PLA reagents (Sigma-Aldrich, St. Louis, MI, USA) as reported previously [25]. Antibodies used were anti-MARCKS (D88D11) XP Rabbit monoclonal antibody (Cell Signaling Technologies, Danvers, MA, USA), Anti-ErbB2/HER2 antibody [3B5] (abcam), and anti-ErbB2 (HER-2) Monoclonal Antibody (2G11), eBioscience (Thermo Fisher Scientific). All images were equally adjusted for brightness and contrast for clarity.

### 2.7. shRNA-Expressing Lentivirus Production and Infection

Lentivirus expressing shLuc and shMARCKS was generated using pGreenPuro vectors (System Biosciences, Mountain View, CA, USA) and HEK293T cells. SK-BR-3 cells were infected with lentiviral particles in the presence of polybrene. One day after infection, the cells were selected with puromycin to allow endogenous protein knockdown. shRNA target sequences for each genes are as follows; shLuc, 5′-CGTGCGTTGTTAGTACTAATCCTATTT-3′; shMARCKS, 5′-GCTTTGACCAATTTGACTTAG-3′.

### 2.8. Cell Proliferation Assay

Cell Proliferation was estimated using the cell counting kit-8 (CCK-8) assay and BrdU incorporation assay. The CCK-8 assay was performed using CCK-8 reagents (Dojindo Molecular Technologies, Inc., Kumamoto, Japan) according to the manufacturer’s instructions. SK-BR-3 cells were seeded in 96-well plates. After 24 h incubation, CCK-8 solution was added to measure cell viability. After 1 h incubation at 37 °C in 5% CO_2_, the absorbance of each well was measured at 450 nm.

### 2.9. TCGA Data Extraction and Analysis

Clinical and gene expression data for breast cancer patients were obtained from the Breast Invasive Carcinoma (TCGA, Firehose Legacy) dataset using the cBio Cancer Genomics Portal (http://cbioportal.org (accessed on 25 July 2025)) [28]. HER2 status (positive/negative) was defined according to clinical standards. HER2 positivity was determined as either an IHC score of 3+ or an IHC score of 2+ with confirmed gene amplification by fluorescence in situ hybridization (FISH). Patients were stratified into two groups based on MARCKS mRNA expression Z-scores relative to all samples: MARCKS high (z-score > 1, *n* = 166) and MARCKS low (z-score < −1, *n* = 170).

To explore clinical correlations, we examined associations between MARCKS expression levels and key clinical features, including ER and PR status (IHC), tumor mutation burden (TMB), and age at diagnosis. ER and PR positivity were treated as binary variables (1 for positive, 0 for negative) and analyzed as categorical data. TMB values were extracted directly from the TCGA dataset and compared between MARCKS high and low groups, while age at diagnosis was treated as a continuous variable.

Kaplan–Meier survival analysis was performed to assess overall survival (OS) differences between the MARCKS high and low expression groups within the HER2 positive cohort. Statistical significance was determined using the log-rank test.

To evaluate whether the associations between MARCKS expression and clinicopathological parameters were independent, we performed univariate and multivariate logistic regression analyses using JMP (Student Edition 18.2.1). The variables included were ER status, PR status, HER2 status, TMB (nonsynonymous), and age at diagnosis.

### 2.10. Immunohistochemistry

Serial sections (3 μm) were prepared using 10% formalin-fixed and paraffin-embedded tissue from the same cases used for RIME. PATHWAY anti-HER-2/neu (4B5) Rabbit Monoclonal Primary Antibody (Roche, Basel, Switzerland) was used for IHC of HER2. HER2 staining was performed using an ultraView Universal DAB Detection Kit (Roche) on a BenchMark ULTRA automated stainer (Roche). IHC of MARCKS was used MARCKS (D88D11) XP Rabbit monoclonal antibody (Cell Signaling Technologie). MARKKS IHC was performed manually using the Dako EnVision FLEX (Agilent Technologies, Santa Clara, CA, USA). For both HER2 and MARCKS staining, the antigen-antibody complexes were visualized with 3,3′-diaminobenzidine solution (1 mmol/L 3,3′-diaminobenzidine) reagent and counterstained with hematoxylin.

## 3. Results

### 3.1. Optimization of HER2 RIME in Cultured Cells

To test whether RIME technique can be applied to membrane proteins like HER2, we first evaluated HER2 RIME using SK-BR-3 human breast cancer cell lines, which are known for their high expression level of HER2 protein. While nuclear protein extracts were selectively prepared in the original RIME protocol, we used an isotonic buffer containing 0.5% (*v*/*v*) NP-40 and 0.25% (*v*/*v*) Triton X-100 to collect the cytosolic fraction, including membrane protein for HER2 RIME experiments (Supplementary Figure S1A). From a pretest using 3 different antibodies against HER2 (DAKO A0485, Abcam, ab16901, and Trastuzumab), we selected Abcam anti-HER2 monoclonal antibody (ab16901) for further RIME experiments using human specimens because of its high coverage and abundant co-precipitation of known interacting proteins (Supplementary Figure S1B–D).

### 3.2. Identification of HER2 Interactome from Breast Cancer Specimens

**Figure 1:**
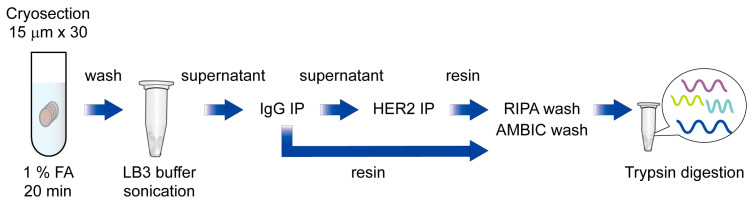
Schematic diagram of HER2 RIME using breast cancer specimens.

Next, to test whether RIME technique could be applied to capture HER2 interactome from human breast cancer specimens, we conducted an HER2 RIME experiment using a HER2 strong positive and ER weak positive human breast cancer specimen (Case 1; HER2 3+, ER TS3). In total, 30 slices of cryosections (15 μm) of tumor were crosslinked in 1% formaldehyde for 20 min, and then proteins were extracted using above mentioned isotonic buffer containing nonionic detergent, and then subjected to further RIME procedure. The supernatant of IgG treated samples was then immunoprecipitated with anti-HER2 antibodies and trypsin-digested after vigorous washing with RIPA and ammonium bicarbonate (AMBIC) buffer. Peptides were cleaned with a detergent removal column and a C18 desalting column, followed by LC-MS/MS analysis (Figure 1).

Briefly, cryosectioned clinical samples were fixed with 1% formaldehyde and washed with cold PBS and resuspended in LB3 buffer, followed by sonication. Next, samples were centrifuged, and supernatants were subjected to IgG and HER2 immunoprecipitation. After an intense wash with RIPA and AMBIC buffer, immunoprecipitated proteins were trypsin-digested and subjected to LC-MS/MS analysis.

**Figure 2:**
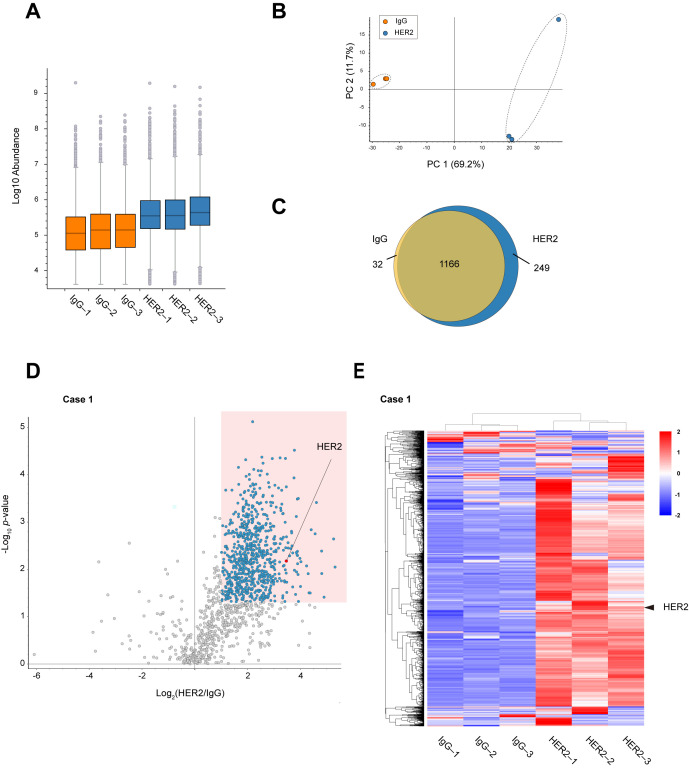
Identification of HER2 interactome from breast cancer specimens. (**A**) Peptide abundance in each RIME sample (IgG 1–3 and HER2 13). The vertical axis is displayed on a logarithmic scale. The lower portion of each box represents the second quartile, while the upper portion represents the third quartile. Gray circles outside the whiskers indicate outliers. (**B**) Principal component analysis (PCA) plot showing the distribution of identified proteins in each RIME sample (IgG 1–3 and HER2 1–3). (**C**) Venn diagram showing the overlap and uniquely identified proteins between IgG and HER2 RIME groups. (**D**) Volcano plot summarizing the label-free quantitative results of HER2 RIME in the human breast cancer specimen Case 1. Proteins identified as statistically significant (fold change >2, *p*-value < 0.05, *n* = 3) are highlighted in blue. HER2 is indicated by a red circle. (**E**) Heatmap showing the proteome profiles of identified proteins. Each row represents a protein, and each column represents a RIME sample. Normalized z-scores of protein abundance are depicted using a color scale relative to expression level of each protein. The hierarchical clustering results are displayed on the left side. HER2 protein is marked with arrowheads.

Comparison of peptide abundance indicated that HER2 RIME showed peptides enrichment much more than IgG RIME control (Figure 2A). Principal component analysis (PCA) was performed to evaluate the protein profiles identified using IgG and HER2 RIME. The PCA plot demonstrates a clear distinction between the two groups, with samples clustered based on the antibody used (Figure 2B). A Venn diagram of identified proteins also revealed specific proteins were identified in HER2 RIME groups (Figure 2C). Label-free quantification identified 719 proteins as significantly enriched proteins in HER2 RIME (Fold Change (HER2/IgG) >2, *p*-value < 0.05) (Figure 2D). These enriched protein groups expectedly included HER2 protein with high significance, indicating successful HER2 RIME from human specimen (Figure 2E, Supplementary Table S1). To further assess technical reproducibility, Pearson correlation coefficients were calculated for the proteomic profiles across the three HER2 RIME replicates. High correlation coefficients (r = 0.93) confirmed robust reproducibility and suggest that the observed variation mainly reflects biological heterogeneity within the tumor tissue rather than technical noise (Supplementary Figure S2A).

**Figure 3:**
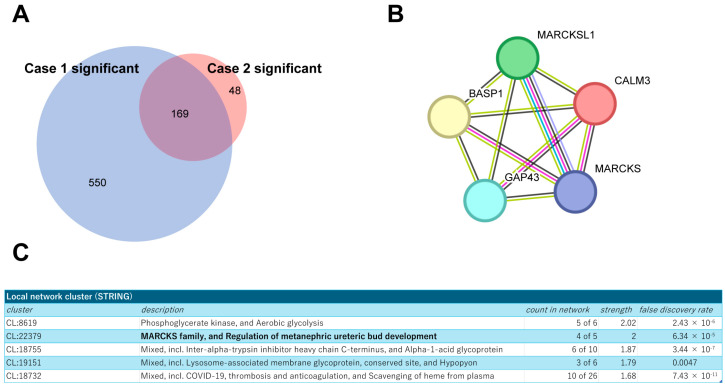
Profiling of the commonly identified proteins from both tumor samples. (**A**) Venn diagram showing the overlap of identified proteins between two breast cancer specimens, Cases 1 and 2. (**B**) Schematic representation of the MARCKS protein complex network generated using the STRING database. (**C**) Results of local network cluster analysis of commonly identified proteins using STRING.

Next, building on these findings, we extended our analysis to a secondary pathological specimen (Case 2; HER2 3+, ER TS3, Supplementary Figure S2B) to refine the interactome by comparing the identified proteins from both sources. This comparative approach allowed us to focus on 169 proteins that were consistently co-identified with HER2 in both specimens (Figure 3A), suggesting their potential significance in the HER2 protein complex. The STRING protein interaction database (https://string-db.org/ (accessed on 12 April 2024)) was used to place them into network contexts and search them for functionally enriched processes/pathways and local network clusters. Among them, the local network cluster indicated enrichment of Myristoylated Alanine-Rich C Kinase Substrate (MARCKS) protein complex components (Figure 3B,C). As functional and physical interactions between HER2 and MARCKS protein have been scarcely explored, we then focused on the cluster of these proteins for further analysis.

### 3.3. Clinical Relevance of MARCKS Expression in HER2-Positive Tumors

MARCKS is a membrane-associated protein that plays a key role in actin cytoskeletal regulation, vesicle trafficking, and cell motility. It is a major substrate of protein kinase C (PKC) and has been implicated in various oncogenic processes, including tumor progression and metastasis [29]. MARCKS also modulates PI3K/AKT and MAPK signaling pathways [30], which are critical for cancer cell survival and proliferation, and have been implicated in aggressive breast cancer subtypes [31,32].

**Figure 4:**
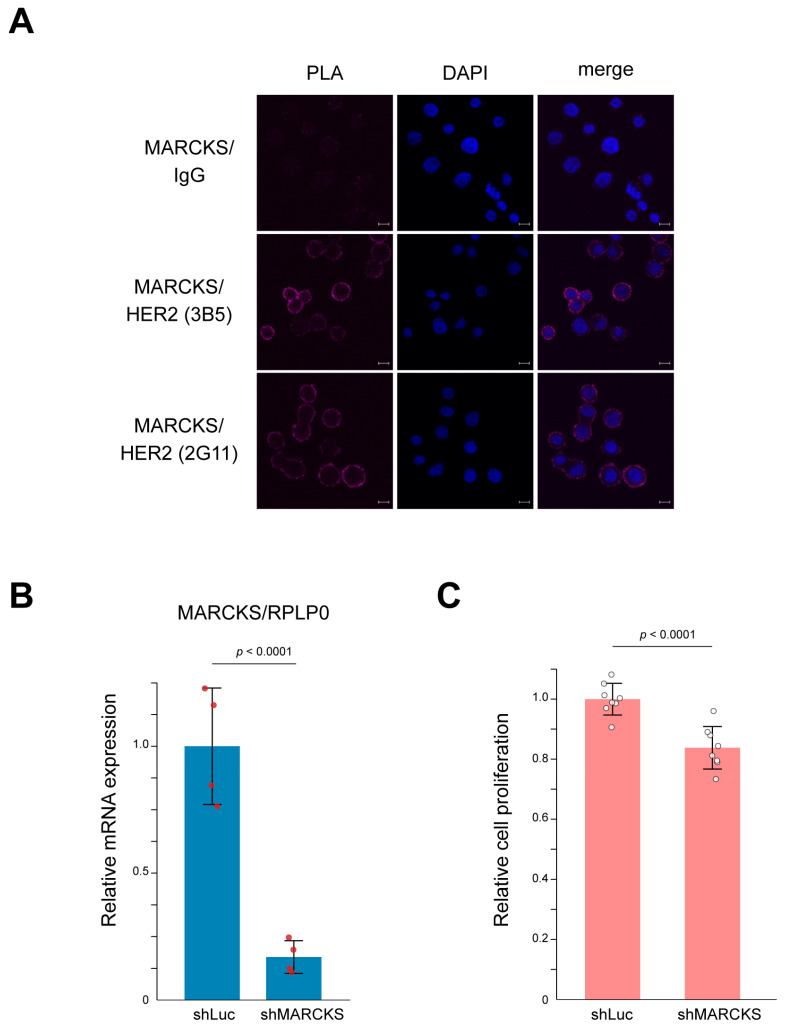
Interaction between HER2 and MARCKS and its functional significance in SK-BR-3 cells. (**A**) Representative images of the proximity ligation assay (PLA) confirming the membrane interaction between HER2 and MARCKS in SK-BR-3 cells. A mouse IgG was used as a negative control. The interaction was validated using two independent HER2 antibodies (3B5 and 2G11). Nuclei were counterstained with DAPI (blue). Scale bars represent 10 μm. (**B**) Establishment of MARCKS knockdown cells using lentiviral shRNA targeting MARCKS. Knockdown efficiency was validated by qPCR. Cells expressing shRNA against luciferase (shLuc) were used as a control. *n* = 4; *p*-value is indicated in the graph. (**C**) CCK-8 assay demonstrating that MARCKS knockdown significantly suppressed the proliferation of SK-BR-3 cells compared to control (shLuc-expressing) cells. *n* = 8; *p*-value is indicated in the graph.

To directly confirm the interaction between HER2 and MARCKS, we first performed a proximity ligation assay (PLA) using the HER2-positive breast cancer cell line SK-BR-3. As shown in Figure 4A, PLA signals indicated a clear membrane interaction between HER2 and MARCKS. In contrast, the HER2-negative breast cancer cell line MCF-7 showed no detectable PLA signals (Supplementary Figure S3), supporting the specificity of the HER2–MARCKS interaction. SK-BR-3 cells are well characterized as HER2-amplified breast cancer cells whose proliferation is strongly dependent on HER2 signaling pathways [33,34,35]. To further investigate the functional relevance of this interaction, we established MARCKS knockdown cells by infecting SK-BR-3 cells with a lentivirus expressing shRNA targeting MARCKS. Successful knockdown was confirmed by quantitative PCR (Figure 4B). Cell counting kit-8 (CCK-8) assays demonstrated that MARCKS knockdown significantly suppressed cell proliferation compared with control cells (Figure 4C). Together, these results indicate that MARCKS functions as a novel HER2-interacting protein that contributes to key cellular phenotypes such as proliferation in HER2-positive breast cancer cells.

**Figure 5:**
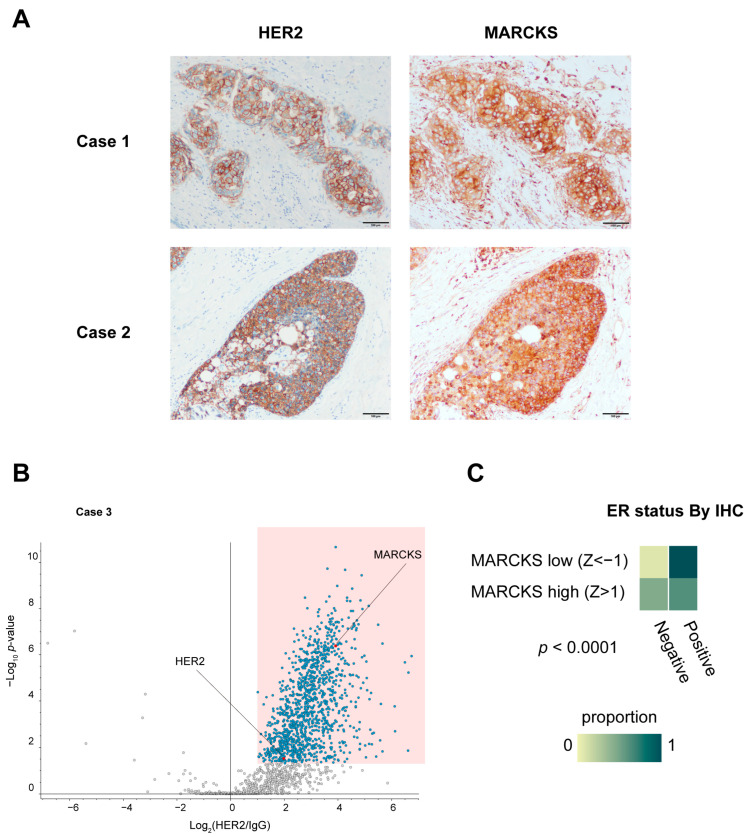
Immunohistochemical validation and clinical relevance of MARCKS expression in breast cancer. (**A**) Immunohistochemical staining of HER2 and MARCKS in breast cancer specimens used for RIME analysis (Cases 1 and 2). All sections were obtained from the same continuous specimen, and images were acquired at 40× magnification. Scale bars represent 100 μm. (**B**) Volcano plot of HER2 RIME profiling performed on an additional HR-negative, HER2-positive breast cancer specimen (Case 3), showing that MARCKS was again identified as a HER2-interacting protein under HR-negative conditions. (**C**) Heatmap showing the proportion of ER-positive and ER-negative cases based on immunohistochemistry (IHC) in breast cancer samples from the TCGA Breast Invasive Carcinoma (Firehose Legacy) dataset. Samples were classified into MARCKS high (z-score > 1) and MARCKS low (z-score < –1) groups according to RNA-seq data. ER positivity was coded as 1 (positive) or 0 (negative). The difference between groups was statistically significant (*p* < 0.0001).

To corroborate the direct interaction observed in vitro, we also evaluated the co-localization of HER2 and MARCKS proteins in HER2 3+ human breast cancer specimens (Case 1 and Case 2) by immunohistochemistry. These analyses confirmed membrane localization of MARCKS in carcinoma cells where HER2 was strongly expressed (Figure 5A), supporting our in vitro PLA results. MARCKS immunoreactivity was also detected in inflammatory cells and fibroblasts surrounding the carcinoma cells, where HER2 expression was absent. To further explore the clinical implications of the HER2–MARCKS interaction, we conducted additional RIME profiling using an HR-negative, HER2-positive tumor specimen (Case 3), which confirmed that MARCKS was consistently identified as a HER2 interactor even in the HR-negative context (Figure 5B). This finding suggests that the HER2–MARCKS interaction extends beyond HR-positive tumors and may also be relevant in more aggressive HR-negative HER2-positive subtypes.

We next analyzed the Cancer Genome Atlas (TCGA) dataset to investigate broader clinical correlations of MARCKS expression. Univeriate analysis indicated that high MARCKS expression (mRNA z-score > 1) was significantly associated with HER2 positivity (*p* = 0.0004), ER and PR negativity in IHC data (both *p* < 0.0001), higher nonsynonymous tumor mutation burden (*p* = 0.0019), and younger age at diagnosis (*p* < 0.0001) (Figure 5C and Supplementary Figure S4A–C). To determine whether these associations were independent, we performed a multivariate logistic regression analysis including HER2 status, ER status, PR status, TMB, and Diagnosis Age as covariates. This analysis showed that both HER2 positivity (OR 2.969, *p* = 0.0074) and ER negativity(OR 0.20, *p* = 0.0028) remained independent predictors of high MARCKS expression. Diagnosis Age also retained significance with a modest effect size (*p* = 0.0330), while PR status, TMB did not remain significant (Table 1). Although high MARCKS expression correlated with parameters associated with more aggressive disease features, no significant difference in overall survival was observed between the high and low MARCKS expression groups within the HER2 positive cohort, highlighting the need for further studies in larger, stratified cohorts (Supplementary Figure S5).

Altogether, these results indicated that the RIME method can be applied to examine protein-protein interactions of membrane proteins like HER2 using human specimens and has the potential for identifying novel clinical markers based on protein-protein interactions.

## 4. Discussion

Here, we established the HER2 RIME method for analyzing HER2-associated protein complexes in human breast cancer specimens. In this approach, we modified the original RIME protocol, which was developed to examine the transcriptional protein complexes in the cell nucleus, to investigate proteins enriched in cytosolic and membrane fractions. By integrating immunoprecipitation using formalin-fixed cytosolic and membrane fractions with label-free quantitative mass spectrometry, we successfully identified HER2-interacting partners from human clinical samples. Although tissue-based proteomic analyses are often challenged by high background levels, our approach enabled the identification of reliable HER2-interacting proteins with high sensitivity and reproducibility.

Although several studies have explored the interactome of the ERBB family [20,21,36], including HER2, most were limited to tumor-derived cultured cell lines. In this study, we conducted RIME analysis on actual clinical breast cancer specimens, enabling the identification of HER2-associated proteins that better reflect in vivo tumor biology. This approach not only provides a more physiologically relevant insight into the HER2 interactome but also offers broader applications for investigating other oncogenic pathways in clinical samples.

Among the identified HER2-interacting proteins, we focused on the MARCKS protein, which had not been previously linked to HER2 biology. MARCKS, a substrate of protein kinase C (PKC), is a myristoylated membrane protein that binds to PIP2 at the plasma membrane [37,38]. Upon phosphorylation, MARCKS associates with actin filaments, regulating cell motility and division, while also enhancing PIP3 production via PI3K, thereby activating proliferative signaling pathways such as the AKT pathway [37]. Although MARCKS activation has been implicated in the aggressive behavior of certain breast cancer subtypes, including inflammatory breast cancer (IBC) [30,31,32], its complex formation with HER2 had not been previously identified. Our analysis revealed that MARCKS forms a complex with HER2, and MARCKS knockdown resulted in a statistically significant but modest reduction of cell proliferation in SK-BR-3 cells (Figure 4C). However, this effect was limited, suggesting that MARCKS may have additional functional contributions to HER2 signaling, such as involvement in cell motility, which warrant further investigation. Additional important, MARCKS expression showed a negative correlation with ER and PR expression. This finding aligns with the observation that aggressive breast cancer subtypes, such as IBC, are often HER2-positive and hormone-insensitive (ER/PR-low), suggesting that MARCKS may contribute to the hormone receptor-negative phenotype observed in these tumors. Consistently, we also identified MARCKS as a HER2 interactor in an additional HR-negative, HER2-positive tumor specimen (Case 3), supporting its potential relevance in more aggressive, hormone receptor-negative disease contexts. However, these preliminary results are based on a limited number of clinical samples (*n* = 3) and should therefore be interpreted with caution; validation in larger cohorts will be necessary to confirm these observations.

In HER2 positive patients, our TCGA analysis confirmed that high MARCKS expression was significantly associated with HER2 positivity, ER and PR negativity, higher tumor mutational burden, and younger age at diagnosis. Multivariate logistic regression further demonstrated that HER2 positivity, ER negativity, and younger diagnosis age remained independently associated with high MARCKS expression, while PR status and TMB did not retain independent significance after adjustment. Although these findings suggest that MARCKS expression may be linked to features of hormone receptor insensitivity and tumor biology, Kaplan–Meier survival analysis did not reveal a significant difference in overall survival between MARCKS high and low groups within HER2 positive patients. This discrepancy may be partly due to the limited statistical power and cohort heterogeneity resulting from HER2 IHC-based stratification. Therefore, larger, well-stratified cohorts will be required to determine whether MARCKS expression has independent prognostic value within distinct HR/HER2 subtypes. Additionally, the presence of MARCKS expression in stromal and inflammatory cells, as observed by immunohistochemistry (Figure 5A), could have influenced the bulk tissue correlations with clinical features. Taken together, these data highlight the potential clinical relevance of MARCKS in HER2-positive breast cancer, but larger, well-stratified studies will be necessary to validate its prognostic and therapeutic implications more robustly.

In addition to HER2-positive breast cancer, increasing attention has been given to HER2-low breast cancer, a newly recognized category characterized by HER2 IHC scores of 1+ or 2+ without gene amplification [39,40]. Recent advances in HER2-targeted antibody-drug conjugates (ADCs), such as trastuzumab deruxtecan (T-DXd), have demonstrated clinical efficacy in HER2-low breast cancer, underscoring the need for a deeper understanding of HER2 signaling in this subtype [41]. However, the molecular mechanisms governing HER2 signaling in HER2-low tumors remain poorly understood. Given that the HER2 RIME method successfully identified HER2 interactors in clinical HER2-positive specimens, it may also serve as a valuable tool for elucidating the HER2 interactome in HER2-low breast cancer. Investigating how HER2-associated protein complexes differ between HER2-low and HER2-positive tumors may provide insights into their distinct biological behaviors and therapeutic vulnerabilities.

Taken together, the HER2 RIME method established in this study offers a robust, high-throughput approach for identifying protein complexes in clinical specimens. By applying this method to larger sample cohorts, it may be possible to identify precise biomarkers based on protein complex formation. While this study focused on HER2 in breast cancer, the approach holds significant potential for broader applications in analyzing protein interactomes across various cancer types and target molecules. This method may facilitate the discovery of novel biomarkers and contribute to the development of targeted treatment strategies beyond HER2-positive breast cancer.

## 5. Conclusions

In this study, we successfully applied the RIME (Rapid Immunoprecipitation Mass Spectrometry of Endogenous Proteins) technique to analyze the HER2 interactome using human breast cancer specimens. By optimizing the protocol to accommodate membrane proteins, we identified multiple HER2-associated proteins, including the novel interaction partner MARCKS. Our findings suggest that MARCKS expression correlates with ER negativity, implicating its potential role in aggressive tumor biology and serving as a potential prognostic indicator. However, these conclusions are based on a limited number of clinical samples and functional assays, and should therefore be interpreted with caution until validated in larger, independent cohorts.

This work demonstrates the feasibility and versatility of using RIME for studying protein–protein interactions in clinical tissue samples, including membrane-bound receptors like HER2. The method can serve as a powerful platform for uncovering clinically relevant interactomes and identifying potential prognostic and diagnostic biomarkers that characterize disease progression and may guide treatment stratification. Further studies applying HER2 RIME to a broader cohort, including HER2-low breast cancer, may lead to the development of novel diagnostic and prognostic tools, and potentially predictive tools, for precision oncology.

## Figures and Tables

**Table 1:** Univariate and multivariate logistic regression analysis for factors associated with high MARCKS expression.

Variable	Univariate	Multivariate	
	*p*-Value	*p*-Value	Odds Ratio (95% CI)
HER2 status by ICH (+/−)	**0.0004**	**0.0074**	2.969 (1.35–6.70)
ER status by ICH (+/−)	**<0.0001**	**0.0028**	0.203 (0.068–0.56)
PR status by ICH (+/−)	**<0.0001**	0.1307	0.519 (0.22–1.23)
TMB (nonsynonymous)	**0.00019**	0.0812	1.274 (0.99–1.71)
Diagnosis Age	**<0.0001**	**0.0330**	0.973 (0.94–0.99)

Values in bold indicate statistically significant differences (*p*-Value < 0.05).

## Data Availability

The raw proteomic data supporting the findings of this study are publicly available in the jPOST repository under the accession numbers JPST003490, JPST003671, and JPST003937.

## Supplementary Materials

The following supporting information can be downloaded at: https://www.mdpi.com/article/10.3390/cancers17172882/s1, Supplementary Figure S1: Identified peptides from HER2 RIME using SK-BR-3 cells; Supplementary Figure S2: Assessment of reproducibility for HER2 RIME samples; Supplementary Figure S3: Negative control for PLA using HER2-negative breast cancer cell line MCF-7; Supplementary Figure S4: TCGA analysis of PR status, tumor mutational burden, and age at diagnosis in relation to MARCKS expression; Supplementary Figure S5: Kaplan–Meier analysis of overall survival based on MARCKS expression in HER2 positive breast cancer samples from TCGA; Table S1: List of significantly enriched proteins identified by HER2 RIME in Case 1 breast cancer specimen.

## Abbreviations

The following abbreviations are used in this manuscript:

ADC	Antibody–Drug Conjugate
AGC	Automatic Gain Control
AMBIC	Ammonium Bicarbonate
ER	Estrogen Receptor
EGFR	Epidermal Growth Factor Receptor
FA	Formaldehyde
FDR	False Discovery Rate
HER2	Human Epidermal Growth Factor Receptor 2
IHC	Immunohistochemistry
IP	Immunoprecipitation
ISH	In Situ Hybridization
LC-MS/MS	Liquid Chromatography-Tandem Mass Spectrometry
MARCKS	Myristoylated Alanine-Rich C Kinase Substrate
MW	Molecular Weight
PBS	Phosphate-Buffered Saline
PI3K	Phosphatidylinositol 3-Kinase
PR	Progesterone Receptor
RIME	Rapid Immunoprecipitation Mass Spectrometry of Endogenous Proteins
T-DXd	Trastuzumab Deruxtecan
TCGA	The Cancer Genome Atlas
TMB	Tumor Mutation Burden
