# Supplementary material for: HER2 Interactome Profiling Reveals MARCKS as a Candidate Marker Associated with Aggressive Breast Cancer
Source: Cancers (Basel). 2025 Sep 2;17(17):2882. doi: 10.3390/cancers17172882 (PMC12428157; doi:10.3390/cancers17172882)
Supplement: Supplementary file 1 [file cancers-17-02882-s001.zip › Yokoyama et al_cancers_Supplemental Figure.pdf]

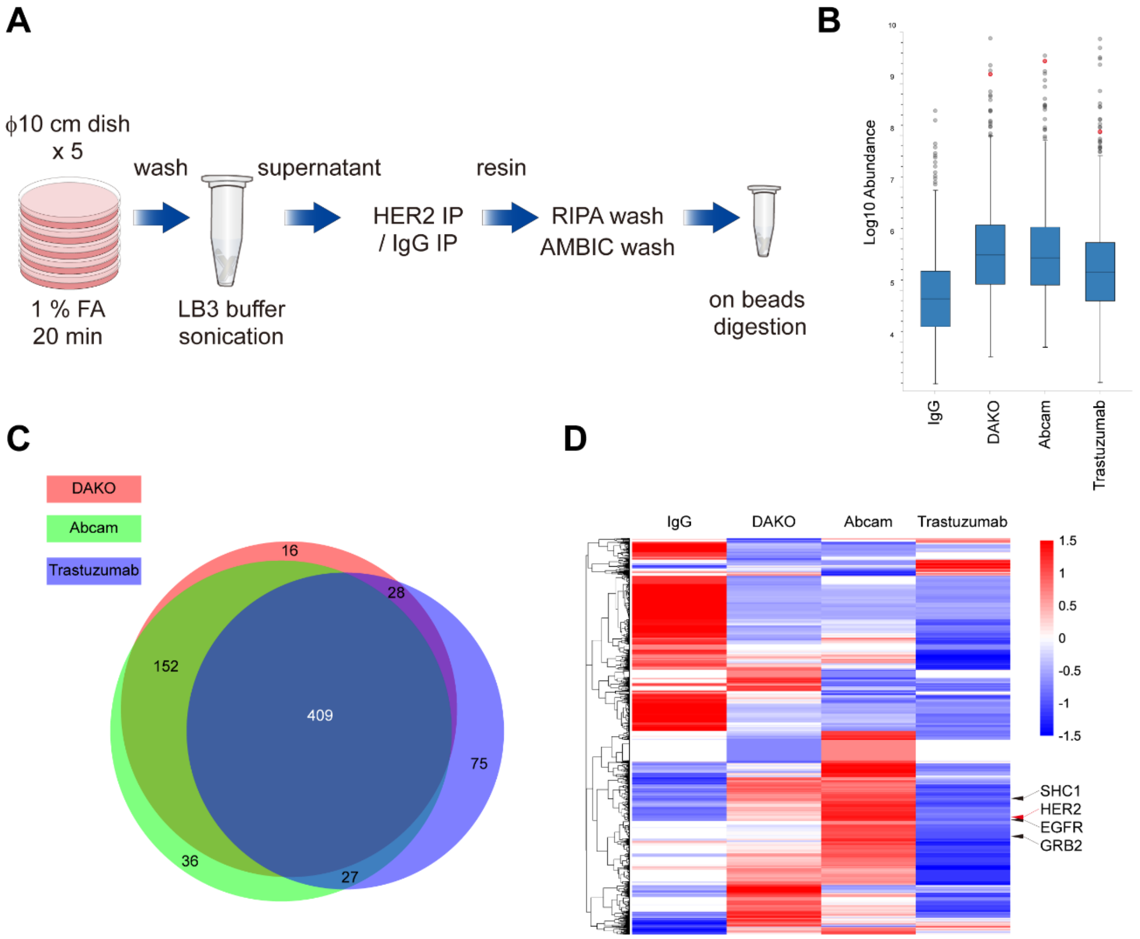

Supplementary Figure S1. Identified peptides from HER2 RIME using SK-BR-3 cells.

A. SK-BR-3 cells were fixed with 1 % of formaldehyde and washed with cold PBS and resuspended in LB3 buffer, followed by sonication. Samples were then centrifuged, and supernatants were subjected to IgG or HER2 immunoprecipitation. After intense wash with RIPA and AMBIC buffer, immunoprecipitated proteins were trypsin-digested and subjected to LC-MS/MS analysis. B. Peptide abundance in each RIME sample (IgG, DAKO, Abcam, and Trastuzumab). The vertical axis is displayed on a logarithmic scale. The lower portion of each box represents the second quartile, while the upper portion represents the third quartile. Gray circles outside the whiskers indicate outliers, and red circles represent HER2 peptides. C. Venn diagram showing the overlap and uniquely identified proteins between IgG and the three different antibodies used for HER2 RIME. D. Heatmap showing the proteome profiles of identified proteins. Each row represents a protein, and each column represents a RIME sample. Normalized z-scores of peptide abundance are depicted using a color scale relative to the quantified signal intensity of each protein. The hierarchical clustering results are displayed on the left side. HER2 and known interacting proteins (SHC1, EGFR, and GRB2) are marked with arrowheads.

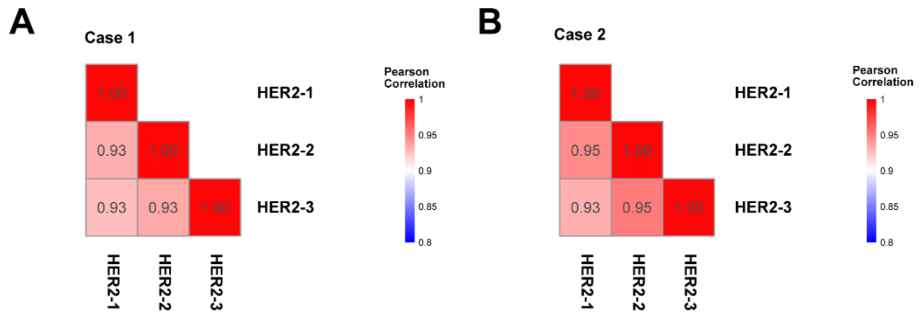

Supplementary Figure S2. Assessment of reproducibility for HER2 RIME samples.

A. Pearson correlation coefficients among the three technical replicates from Case 1, calculated using log2-transformed protein abundance values, and visualized as a heatmap with a color scale indicating the degree of correlation (blue to red). B. Pearson correlation coefficients among the three technical replicates from Case 2, calculated using log2-transformed protein abundance values, and visualized as a heatmap with a color scale indicating the degree of correlation (blue to red). High correlation coefficients in both cases confirm the technical reproducibility of the HER2 RIME proteomic profiles.

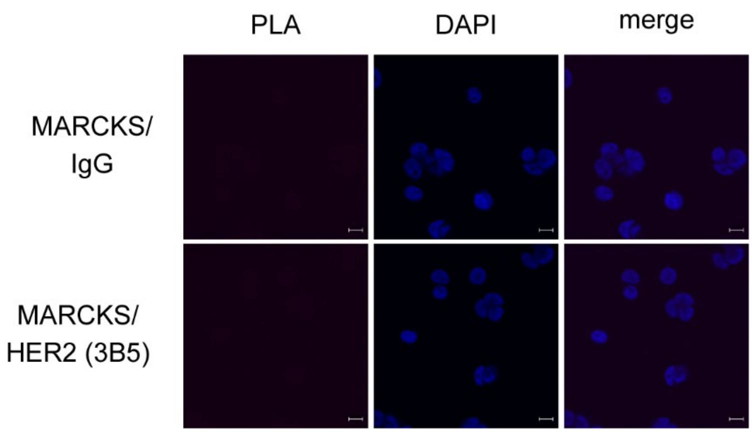

Supplementary Figure S3. Negative control for PLA using HER2-negative breast cancer cell line MCF-7.

Representative images of the proximity ligation assay (PLA) performed in HER2-negative MCF-7 cells. No specific PLA signals for the HER2–MARCKS interaction were detected, confirming the absence of HER2-dependent interaction in this cell line. A mouse IgG was used as an additional negative control for antibody specificity. Nuclei were counterstained with DAPI (blue). Scale bars represent 10 μm.

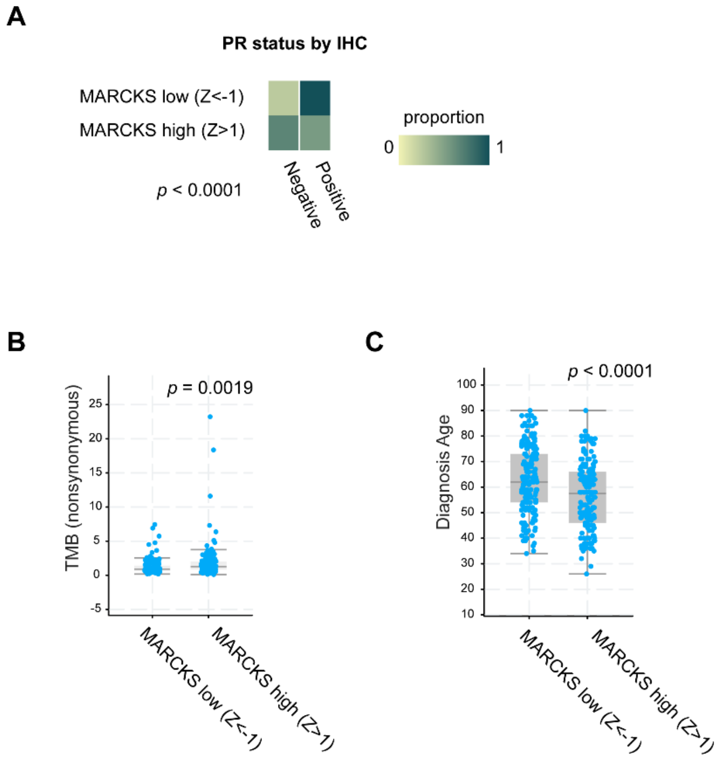

Supplementary Figure S4. TCGA analysis of PR status, tumor mutational burden, and age at diagnosis in relation to MARCKS expression.

A. Comparison of PR status between MARCKS high and MARCKS low groups, showing a significantly higher frequency of PR negativity in the MARCKS high group ($p < 0.0001$). B. Comparison of tumor mutational burden (TMB) between MARCKS high and MARCKS low groups, indicating a trend toward higher TMB in the MARCKS high group ($p = 0.0019$). C. Comparison of age at diagnosis between MARCKS high and MARCKS low groups, showing that patients in the MARCKS high group were diagnosed at a significantly younger age ($p < 0.0001$).

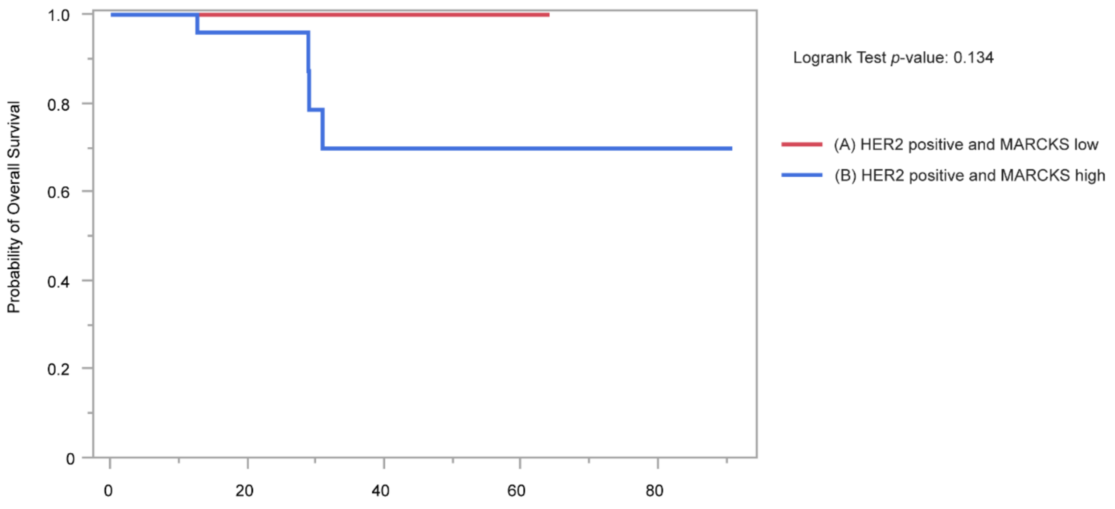

Supplementary Figure S5. Kaplan–Meier analysis of overall survival based on MARCKS expression in HER2 positive breast cancer samples from TCGA.

Kaplan–Meier survival curves comparing overall survival between MARCKS high (z-score > 1, n = 33) and MARCKS low (z-score < –1, n = 15) groups in HER2 positive breast cancer samples from the Breast Invasive Carcinoma (TCGA, Firehose Legacy) dataset. No significant difference in overall survival was observed between the two groups (*p* = 0.134, log-rank test).
